# Loneliness in Young People with ADHD: A Systematic Review and Meta-Analysis

**DOI:** 10.1177/10870547241229096

**Published:** 2024-02-23

**Authors:** Angelina Jong, Clarissa Mary Odoi, Jennifer Lau, Matthew J.Hollocks

**Affiliations:** 1 King’s College London Institute of Psychiatry Psychology & Neuroscience, London, UK; 2 Queen Mary University of London Wolfson Institute of Population Health, London, UK

**Keywords:** ADHD, youth, psychosocial functioning, mental health, meta-analysis

## Abstract

Many studies focus on problematic peer functioning in attention deficit/hyperactivity disorder (ADHD) but loneliness has been studied less. This paper examined (1) The loneliness level differences between young people (below 25 years old) with ADHD and those without ADHD, and (2) The association between loneliness and mental health difficulties in young people with ADHD. Six electronic databases were searched and 20 studies were included. A random effects meta-analysis was carried out in RStudio using the metafor package for the first question, while a narrative synthesis summarized the findings for the second question. The meta-analysis (*n* = 15) found that young people with ADHD reported significantly higher loneliness than those without ADHD, with a small-to-medium weighted pool effect (Hedges’ *g* = 0.41) and high heterogeneity (*I*^2^ = 75.1%). For the second question (*n* = 8), associations between loneliness and mental health difficulties in ADHD was found (*r* = 0.05–0.68). Targeted research and interventions on loneliness in young people with ADHD is needed.

## Introduction

Attention-deficit/hyperactivity disorder (ADHD) is a common neurodevelopmental disorder, with an estimated prevalence of between 5% and 13% in children and adolescence (Polanczyk et al., 2007; Thomas et al., 2015; Willcutt, 2012). Young people with ADHD tend to experience difficulties in their social functioning and peer relationships (Hoza et al., 2005; McQuade & Hoza, 2008). Many studies looking at social-emotional functioning in ADHD focus on social networks and peer functioning problems, with fewer research on young people’s experience of and satisfaction with their relationships. This is an important distinction as loneliness, or perceived social isolation, is the subjective feeling of distress due to a perceived deficit in the quantity and quality of one’s social relationships (Peplau & Perlman, 1982). An individual may be objectively socially isolated but may not have a negative perception of their relationships and equally, someone may find their social relationships lacking despite having a large social network (Hawkley & Cacioppo, 2010). Thus, loneliness captures the individual’s internal emotional state and is a distressing experience, possibly as a signal to avoid threats to social relationships and to motivate social repair. As loneliness and social isolation (e.g., number of friends or size of social network) have been found to be weakly correlated (Coyle & Dugan, 2012), it is important to capture the loneliness experience of the young person rather than relying on just the objective measures of social isolation.

Loneliness is a public health concern with well-documented adverse effects on mental health (Cacioppo & Cacioppo, 2018; Cacioppo et al., 2002; Christiansen et al., 2021; Eccles et al., 2020), in addition to worsened physical health (Hawkley & Cacioppo, 2010; Holt-Lunstad et al., 2015). In young people in the general population, increasing and chronic loneliness trajectories predict increases in depression, anxiety, self-harm, and suicidal ideation (Heinrich & Gullone, 2006; Ladd & Ettekal, 2013; Qualter et al., 2013; Vanhalst et al., 2013). Given that both loneliness and ADHD are associated with many negative outcomes, it is important to better understand loneliness levels in young people with ADHD to inform future research and clinical directions in this area.

While many young people with ADHD may have difficulties with social functioning, what is still unclear is whether they experience greater loneliness compared to their peers without ADHD. Some find higher levels of loneliness in ADHD compared to their peers (Langher et al., 2009), while others found them not to significantly differ (Heiman, 2005; Houghton et al., 2015). Given the many social-emotional difficulties found in ADHD (Hoza et al., 2005) and research suggesting that loneliness is prevalent in populations with developmental conditions that impact social difficulties in young people, such as in autism spectrum disorder (Bauminger et al., 2003; Hymas et al., 2022; Lasgaard et al., 2010). It is possible that some of these may arise from methodological differences between the studies. Therefore, it would be informative to systematically investigate whether loneliness is elevated in young people with ADHD.

There are factors that may impact the levels of loneliness reported in these studies, thus it would be important to examine potential moderators of loneliness. The demographic makeup of the studies’ samples such as age, ethnicity, and gender may be impacting the loneliness rates. Both levels of loneliness (Mund et al., 2020; Shovestul et al., 2020) and ADHD symptoms tend to change with age (De Rossi et al., 2023). Gender may also influence loneliness in ADHD because males and females with ADHD often present, and are responded to differently (Diamantopoulou et al., 2005). Referral sources may also impact reported loneliness levels as there may be important differences between clinical and community referred samples, such as the degree of impairment in ADHD and presence of comorbidities (Bauermeister et al., 2007). Previous research suggested that the choice of loneliness measure may impact on loneliness levels as there are different dimensions of loneliness (Houghton et al., 2020). Taken together, this suggests that having a clearer understanding of the loneliness levels in young people with ADHD and their possible moderators may be informative.

### Loneliness and Mental Health in ADHD

The high comorbidity with mental health difficulties in ADHD has been well documented: comorbidities can be categorized into both internalizing disorders/behaviors (e.g., anxiety, depression) and externalizing disorders/behaviors (e.g., oppositional defiant disorder, conduct, aggression) (Elia et al., 2008; Jarrett & Ollendick, 2008; S. S. Lee et al., 2011; Ollendick et al., 2008; Pliszka, 2000; Tung et al., 2016). One study demonstrated that not only are loneliness and depression elevated in adolescents with ADHD compared to their peers, but also that loneliness fully mediates the relationship between ADHD diagnosis and depression (Houghton et al., 2020). This suggests that loneliness may play a crucial part in the development of depression in ADHD. As loneliness and mental health difficulties have been demonstrated to have bidirectional relationships in the general population, similar associations may be present in ADHD (Lim et al., 2016; McDowell et al., 2021).

Currently, there does not seem to be a clear understanding of loneliness in young people with ADHD despite the increased risk of loneliness conferred by both the age group (Hawthorne, 2008; Ladd & Ettekal, 2013) and the prevalence of social difficulties (Becker et al., 2012; Elmose & Lasgaard, 2017). It is essential to synthesize findings across different studies and establish whether young people with ADHD are lonelier than their peers. Second, as loneliness has been linked with increased mental health difficulties in the general population (Cacioppo et al., 2006; Hawkley & Cacioppo, 2010; Loades et al., 2020), a better understanding of if, and how, loneliness is associated with mental health in ADHD may shed light on whether loneliness in this population should be targeted in future interventions.

### This Review

In our searches, we have found no similar reviews on loneliness in young people with ADHD. The definition of young person in this review encompasses a mean age of up to 24 years old, in line with the World Health Organization’s definition of young people as between the ages of 10 and 24 (World Health Organization, 2019). This review aims to synthesize findings from studies that explore loneliness in young people with ADHD.

1. Do young people with ADHD experience greater loneliness compared to young people without ADHD?
2. What is the association between loneliness and mental health difficulties (e.g. depression, anxiety, conduct disorder) in young people with ADHD?

If appropriate, meta-analyses will be conducted to address these questions. Further moderator analyses and meta-regressions will also be run, if there are enough studies, to assess the impact of gender, age, loneliness measure, study quality, and recruitment setting on the heterogeneity in the effect sizes.

## Methods

### Search Strategy

A protocol of this review was registered on PROSPERO on February 2, 2022 and adheres to the Preferred Reporting Items for Systematic Reviews and Meta-Analyses (PRISMA) guidance (Page et al., 2021). At the time of registration, there were no other similar systematic reviews or meta-analysis on PROSPERO and the Cochrane library.

The search terms were developed in discussion with researchers knowledgeable in the area of loneliness and neurodevelopmental disorders (JL and MH), and through scoping searches of other ADHD-related and loneliness-related systematic reviews. To identify relevant studies, combinations of the following terms were used: ((“attention deficit hyperactivity disorder”) OR (“attention deficit disorder with hyperactivity”) OR (“attention deficit/hyperactivity”) OR (“attention-deficit/hyperactivity disorder”) OR (ADHD) OR (ADD) OR (ADDH) OR (ADHS) OR (“attention deficit disorder”) OR (TDAH) OR (hyperkine*) OR (“hyperkinetic syndrome”)) AND ((“soc* isolation*”) OR (“subject* isolation*”) OR (“feeling* isolate*”) OR (“lonel*”)). Due to the high frequency of the term “social isolation” in research around peer rejection and exclusion in ADHD or more generally during the COVID pandemic lockdowns, it was decided that the search terms would be limited to specifically perceived social isolation or loneliness in the title, keyword, or abstract. The following databases were searched: PubMed, SCOPUS, PsychINFO APA, Embase, Medline, and Web of Science. The initial search was carried out on February 7, 2022. An updated search was carried out on July 14, 2023. To reduce the risk of missing relevant research, references of included papers and relevant reviews were also manually screened for relevant literature. Results were collated using EndNote library and duplicates were removed before exporting the references (including title and abstract) to Excel for screening. Titles and abstracts were screened according to relevance, with 20% of titles and abstracts also screened by an independent rater. Full-text review of studies likely to meet the inclusion criteria were carried out and 20% of the full-text review was carried out by an independent rater. Any discrepancies in the full-text review were discussed.

### Eligibility Criteria

The inclusion and exclusion criteria for the first research question are listed in Table 1. The inclusion and exclusion criteria for the second research question are similar except that a non-ADHD control group is not required, and a measure of mental health difficulties is required. The definition of intellectual disabilities used in this review follows that of DSM-V, which defines intellectual disabilities as neurodevelopmental disorders that begin in childhood and are characterized by deficits in intellectual functioning and adaptive functioning (American Psychiatric Association [APA], 2013). Participants with learning disorders or difficulties, defined as having difficulties in learning and using specific academic skills than expected for age, schooling and IQ (APA, 2013), are not excluded in this review. For this review, a broad definition of mental health outcomes is used and included any diagnosed psychopathology or symptoms of psychopathology. This includes disorder-specific measures that look at symptoms or presence of diagnosis (e.g., depression), and mental health measures that look at general externalizing or internalizing behaviors symptoms or behaviors.

**Table 1.** Inclusion and Exclusion Criteria for the Primary Review Question.

Inclusion	Exclusion
1. Published in English regardless of date and country 2. Published in peer-reviewed journals 3. Cross sectional or longitudinal quantitative studies 4. Reported participants with a categorical diagnosis of ADHD in accordance with DSM-III, DSM-III-R, DSM-IV, DSM-IV-TR, DSM-5, a categorical diagnosis of hyperkinetic disorder in accordance with ICD-9 or ICD-10 or symptomatic presentation of ADHD (scoring above threshold on ADHD symptom questionnaires with known psychometric properties e.g. ASRS) 5. Reported participants with mean age between 0 and 24 years old 6. Reported at least one self-reported or parent/teacher-reported measure (or subscale) of loneliness (or related concepts such as perceived social isolation e.g., UCLA Loneliness Scale (D. Russell et al., 1980) and Loneliness and Aloneness Scale for Children and Adolescents (Marcoen et al., 1987)	1. Qualitative studies or case studies 2. Participants in the ADHD group or the non-ADHD group had intellectual disabilities and/or other neurodevelopmental conditions such as Autism Spectrum Disorder 3. Studies did not include a non-ADHD control group

### Data Extraction

A data extraction table was created in Excel prior to extraction. The following information was extracted from the studies when available: authors, date, country, objectives, sample size, age and predominant ethnic group of the participants, IQ measure, ADHD measure, loneliness measure, mental health measure, key findings, and results. For multidimensional measures of loneliness, only factors and subscales directly related to loneliness will be extracted; factors and subscales related to other aspects such as aloneness (e.g., positive and negative feelings toward aloneness in The Perth A-loneness Scale (Houghton et al., 2014) will not be extracted. When relevant results or statistics were not reported (such as the mean and standard deviation in comparison studies), authors of the studies were contacted to obtain the analysis. Given the number of studies for each research question, we performed a meta-analysis only for Research Question 1. For the meta-analysis, the means and standard deviations for loneliness in the ADHD groups and non-ADHD comparison groups were extracted from the studies.

### Quality Assessment

The quality of the eligible studies was evaluated using the QualSyst appraisal tool (Kmet et al., 2004). The Qualsyst checklist consists of 14 questions covering different aspects such as the appropriateness of the study design, analytic methods, and so on. The possible summary scores range from 0 to 1, with higher scores indicating higher quality. For this review, we chose a priori not to exclude any papers but to assess the quality and include it in the meta-regression. All eligible studies were rated by two raters and any discrepancies were discussed until consensus was reach.

### Data Analysis

The meta-analysis will only be carried out on the first research question which is to compare loneliness levels in groups of young people with ADHD compared to young people without ADHD. The meta-analysis was conducted using RStudio (R Core Team, 2022) with the “metafor” package (Viechtbauer, 2010). Hedges *g* was chosen as the effect size for the standardized mean difference in this meta-analysis due to its higher accuracy with smaller sample sizes (less than 20). Hedges *g* of 0.2, 0.5, and 0.8 were considered small, medium, and large effect sizes, respectively (Cohen, 1992). A random-effects model will be applied as it assumes that the true effect will differ between studies which may be relevant for the studies included, considering the different types of studies included in this review. To account for differences between the studies (e.g., participant demographics and different loneliness measures), a random-effects model was applied.

For the meta-analysis, if papers report overlapping samples, the paper with the most relevant information will be included in the meta-analysis. For papers reporting separate groups, such as age or ADHD sub-types, they will be combined if possible. If this is not possible, the most relevant or largest group will be included. When studies report multiple loneliness scales/sub-scales, measures the capture peer or perceived social isolation (rather than familial or friendship) will be chosen to increase the validity of the effect size estimates.

For the second research question (association between loneliness and mental health difficulties in young people with ADHD), a meta-analysis was not conducted due to the broad nature and small number of studies included. Instead, a narrative synthesis summarizing the findings of the studies will be conducted. The results will be presented in a table and discussed.

### Heterogeneity

Cochran’s *Q* and *I*^2^ test statistics were carried out to assess heterogeneity and the percentage of variability of effect size due to heterogeneity, respectively. As a smaller number of studies in the meta-analysis can cause Cochran’s *Q* test to have low power, an adjusted alpha level of 0.10 is used. For the *I*^2^ test, the following rough guide will be used: 25% = low heterogeneity, 50% = moderate heterogeneity, 75% = high heterogeneity (Higgins et al., 2003).

### Moderator Analysis

To assess sources of heterogeneity in the meta-analysis, moderator analyses were carried out including subgroup analysis and meta-regression for categorical and continuous variables respectively (Borenstein et al., 2010). Sub-group analysis was carried out when each category had at least five studies. The following characteristics were explored: age and age category, gender (percentage of males), study quality, loneliness measure used, and recruitment setting.

## Results

### Study Methodology and Quality

**Figure 1.**
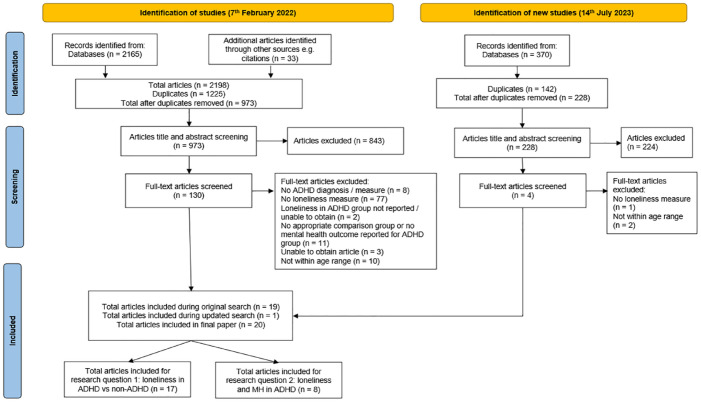
PRISMA flow diagram.

Results from the initial database search on February 7, 2022 (n = 2165) were exported into EndNote Web and duplicates were removed, before exporting the references to Microsoft Excel for title and abstract screening. All titles and abstracts (*n* = 973) were screened according to relevance and 20% of the titles and abstracts were screened by an independent rater, with substantial agreement (*n* = 195, *κ* = 0.78). Full-text review of studies likely to meet the inclusion criteria were carried out (*n* = 130) by the first author and 20% of the full-text review was replicated by the independent rater (*n* = 27, *κ* = 0.88). Any discrepancies in the full-text review were discussed, resulting in 19 studies included. An updated search was carried out on July 14, 2023 and the results were exported into EndNote Web (*n* = 370) and after duplicates were removed, the titles and abstracts of the remaining were screened (*n* = 228) which resulted in the addition of one study. The overall literature search process is summarized in Figure 1 (Moher et al., 2009; Page et al., 2021).

A total of 20 studies are included in this paper: 17 studies included comparisons of levels of loneliness in ADHD versus non-ADHD (Research Question 1). For research question 1, a meta-analysis was carried out and 15 of the 17 studies were included in the meta-analysis. As Houghton, Kyron, Lawrence et al. (2022) and Houghton et al. (2020) had overlapping samples, only Houghton et al. (2020) was included in the meta-analysis because it contained more relevant information (e.g., number of males in the groups). One study was excluded from the meta-analysis as it did not report the mean and standard deviation of the groups (Heiman, 2005). For Research Question 2, eight studies measured the association between loneliness in young people with ADHD and mental health difficulties. Due to the small number of studies investigating different mental health difficulties, a systematic review will be carried out to explore Research Question 2.

In terms of the quality assessment, scores of the papers in this review ranged from 0.64 to 0.91 (see Table S1 in Supplementary Material). Intra-class correlation coefficient showed good agreement between the raters (*κ* = 0.88; 95% CI [0.68, 0.95]). The studies were generally rated highly on clearly reporting their aims, analysis, and results, and had appropriate study designs and analyses for the studies’ aims.

### Systematic Review for Research Question 1: Do Young People With ADHD Experience Greater Loneliness?

#### Research question 1: Study characteristics

Table 2 summarizes the study and sample characteristics of the studies comparing loneliness in ADHD and non-ADHD samples. Studies were conducted across eight countries, predominantly in Western countries and Israel. Besides three studies that were longitudinal (Houghton, Kyron, Lawrence et al., 2022; Matthews et al., 2019; Meinzer et al., 2013), all other studies were cross-sectional in nature.

**Table 2.** Summary of Studies Comparing Loneliness in ADHD and Non-ADHD Group (Research Question 1).

Authors (Year); Country	Sample size *n* (male)	Age *M* (*SD*)	Ethnicity	IQ	Study quality	ADHD measure	Loneliness measure	ADHD loneliness score *M* (*SD*)	n-ADHD loneliness score *M* (*SD*)	Key findings
Al-Yagon (2009); Israel	ADHD-LD: 59 (42) TD: 59 (40)	ADHD-LD: nr TD: nr Overall: 10.05 (1.05)	nr	Average IQ	0.77	Previously diagnosed with both LD and ADHD through psychoeducational and neurological/psychiatric evaluations, according to DSM-IV-TR.	CLSD (Hebrew adaptation)	37.00 (15.16)	26.64 (10.00)	Children with comorbid LD and ADHD reported higher loneliness than children without LD and ADHD, *F*(1,114) = 16.90, *p* < .001.
Al-Yagon (2016); Israel	ADHD-LD: 91 (41) LD: 90 (40) TD: 98 (45)	ADHD-LD: nr LD: nr TD: nr Overall: 15.94 (0.70) no sig. diff between the groups	nr	Normal level, according to WISC-IV	0.82	Previous ADHD diagnosis based on DSM-IV-TR using clinical interview, computerized tests and widely used measures of ADHD symptom. Diagnosis validated by document check.	PNDLS	Peer-network loneliness: 15.09 (5.50) Peer-dyadic loneliness: 13.33 (4.92)	LD; Peer-network loneliness: 14.09 (4.34) Peer-dyadic loneliness: 13.48 (5.45) TD; Peer-network loneliness: 12.87 (3.70) Peer-dyadic loneliness: 11.43 (3.42)	Peer network loneliness: ADHD-LD group reported higher loneliness compared to TD, *F*(2, 277) = 5.67, *p* < .01, η^2^ = .04. No sig. difference between ADHD-LD and LD, and LD and TD. Peer dyadic loneliness: Comparing all 3 groups, *F*(2, 277) = 5.67, *p* < .01, η^2^ = .04. ADHD-LD = LD > TD
Capodieci et al. (2019); Italy	ADHD: 21 (13) N-ADHD-Low-social skills: 21 (13)	ADHD: 9.72 (1.34) N-ADHD-Low-social skills: 9.72 (1.34)	nr	nr	0.68	Informal interviews with teachers and parents; ≥14 on SDAI Controls were matched for control items on the SDAI scale, and sociability on the COM scale	CLSD (Italian adaptation)	83 (10)	Control with weak social skills: 81 (10) Control with normal social skills: nr	There is no difference in loneliness between the ADHD group and the control group with weak social abilities, *F =* 0.15 *p* = .698, η^2^ = .004. No differences were found in loneliness levels between ADHD, N-ADHD with low social skills, and N-ADHD with normal social skills. All children showed a high level of perceived loneliness.
Deckers et al. (2017); Netherlands	ADHD: 76 (54) n-ADHD: 106 (62)	ADHD: Combined = 11.79 (2.48) Child = 9.61 (1.13) Adolescent = 13.75 (1.55) n-ADHD: Combined = 11.61 (2.63) Child = 9.31 (1.26) Adolescent = 14.00 (1.10)	Predominant Caucasian	Excluded if Severe cognitive (i.e., estimated IQ < 70) or language impairments.	0.82	Diagnosed by a multidisciplinary team (interview, psychiatric examination, psychological assessment, observations)	LACA (only relationships with peer subscale was used in the study)	Child: 19.54 (7.98) Adolescent: 15.48 (3.35) Combined: 17.403 (6.302)	Child: 20.32 (6.14) Adolescent: 18.12 (4.58) Combined: 19.241 (5.517)	In the Child group, no sig diff between ADHD or control. In adolescents, sig diff: control > ADHD In ADHD and control group, children reported sig higher levels of loneliness than adolescents.
Elmose and Lasgaard (2017); Denmark	ADHD: 25 (25) n-ADHD: 199 (199)	ADHD: 14.6 (1.04) n-ADHD: 14.1 (0.43)	nr	IQ > 70	0.72	Diagnosis based on ICD-10 criteria for F90 Hyperkinetic disorder and to be without intellectual disability	UCLA (Danish version)	37.6 (7.94)	37.69 (10.23)	No group difference was found in loneliness between adolescents with ADHD and adolescents from regular schools, *p* = ns.
Heiman (2005); Israel	ADHD: 39 (31) n-ADHD: 17 (12)	ADHD: 11.2 (2.05) n-ADHD: 10.2 (1.10)	nr	88 - 120; no profound developmental or psychiatric disorder	0.64	Diagnosed between the age of 5.5 to 10 using: WISC-III, parent interview based on DSM-IV; above 15 on teacher-rating on CRS	CLSD (Hebrew version)	nr	nr	ADHD group not significantly lonelier than n-ADHD group, *p* = ns. Parents and teachers of children with ADHD rated children significantly lonelier than parents, *F*(1, 56) = 16.43, *p* < .001, and teachers, *F*(1, 56) = 20.21; *p* < .001, of children without ADHD.
Houghton et al. (2015); Australia	ADHD: 84 (74) n-ADHD: 84 (73)	ADHD: 15.2 (2.43) n-ADHD: 15.3 (2.49)	63% none 26% Anglo Saxon/European 4% Asian 5% Other	nr	0.82	Previous diagnosis meeting DSM-IV-TR criteria.	PALs	Isolation loneliness: 1.77 (0.12) Friendship loneliness: 4.53 (0.16)	Isolation loneliness: 1.69 (0.11) Friendship loneliness: 4.52 (0.15)	No difference between groups on isolation loneliness, *F*(1, 166) = 1.41, *p* = .236, partial η^2^ = 0.009, and friendship loneliness, *F*(1, 166) = 0.012, *p =* .914, partial η^2^ = 0.001.
Houghton et al. (2020)^a^; Australia	ADHD: 42 (32) Control: 42 (32)	ADHD: 13.01 (2.0) Control was age-matched	nr	nr	0.73	Previously diagnosed by a pediatrician/child psychiatrist as meeting DSM-IV-TR or DSM-5 criteria for ADHD.	PALs	Isolation loneliness: 13.45 (6.71) Friendship loneliness: 23.76 (7.76)	Isolation loneliness: 10.33 (4.26) Friendship loneliness: 28.17 (5.84)	Adolescents with ADHD had lower quality of friendships, *F*(1, 78) = 8.43, *p* = .005, η2 = 0.10, and greater feelings of isolation, *F*(1, 78) = 8.99, *p* = .003, η^2^ = 0.10.
Houghton, Kyron, Lawrence et al. (2022)^a^; Australia	ADHD: 76 (nr) n-ADHD: 238 (131)	ADHD: nr n-ADHD: 13.52 (1.44)	nr	nr	0.77	Previously diagnosed by a pediatrician or child psychiatrist as meeting DSM-IV-TR or DSM-5 criteria for ADHD.	PALs	Isolation loneliness: 11.89 (6.20)^b^	Isolation loneliness: 10.55 (4.93)^b^	Adolescents with ADHD compared to controls, reported higher isolation loneliness (*B =* 2.14, *p =* .009) and lower friendship quality (*B =* 3.03, *p* = .002).
Koutra and Kokaliari (2022); Greece	ADHD: 67 n-ADHD: 295	Overall: 22.5 (5.5)	Overall: 352, 100% Greek	nr	0.77	> 4 on ASRS	UCLA	28.7 (15.7)	20.2 (13.4)	The ADHD group reported significantly higher loneliness scores than the n-ADHD group (*U* = 6,760, *z* = −4.01, *p* < .001)
Langher et al. (2009); Italy	ADHD: 31 (25) No special needs: 31 (25)	ADHD: Primary = 23 Low Secondary = 8 No special needs: Primary = 23 Low Secondary = 8	nr	nr	0.68	Diagnosed by local health department. All hyperactive form.	CLSD	38.52 (14.56)	31.94 (9.71)	Children with ADHD reported greater loneliness than children with no special needs, *F*(1,90) = 4.75, *p* = .03.
Laslo-Roth et al. (2020); Israel	Under 24^b^: ADHD: 34 (6) n-ADHD: 146 (15)	Under 24^b^: ADHD: nr N-ADHD: nr Overall: 22.01 (1.20)	nr	nr	0.82	Previously diagnosed (psychiatrist/psychologist). Documentation confirmed .	UCLA (short version)	1.98^b^ (1.28)	2.03^b^ (0.99)	There were no significant differences found between the loneliness scores of YP with ADHD vs without ADHD, *t*(178) = −0.27, *p =* .78.^b^
Laslo-Roth et al. (2021); Israel	ADHD: 166 (118) n-ADHD: 114 (65)	ADHD: 9.89 (2.20) n-ADHD: 9.33 (2.45)	nr	nr	0.82	Previously diagnosed (psychiatrist/psychologist).	Parent-reported loneliness: “How often does your child seem lonely to you?”	2.93 (1.35)	2.39 (1.09)	Parents of children with ADHD (compared to without) reported their children to have higher levels of loneliness, *F*(3, 276) = 9.07, *p <* .01, Partial ƞ² = .032.
Martin et al. (2019); USA	ADHD: 199 (199) n-ADHD: 74 (74)	ADHD: 9.8 (1.3) n-ADHD: 10.0 (1.3)	ADHD, n-ADHD: Caucasian 83, 65% African American 12, 24% Other/mixed 5, 11%	nr	0.77	Formally diagnosed: DSM-III-R; parent and teacher versions of the DBD and the parent DBD structured interview	CLSD	2.09 (0.83)	1.87 (0.64)	Tests of difference and significance not reported.
Matthews et al. (2019); UK	At age 18, ADHD: 162 (nr) n-ADHD: 1,904 (nr)	At age 18, ADHD: nr n-ADHD: nr Overall: 18.4 (0.36)	nr	Overall: Age 5 on WPPSI-R: 100 (15)	0.86	Past-year diagnosis: DSM-IV or DSM-V criteria	UCLA (Version 3)	2.71 (2.28)	1.47 (1.88)	Tests of difference and significance not reported.
Meinzer et al. (2013); USA	T1: ADHD^b^: 40 (nr) NT: 1,467 (nr) Overall: 1,709 (803)	T1: ADHD: nr NT: nr Overall: 16.6 (1.2)	T1: 62% Black, 36% White, 1% Hispanic, 1% ‘other’	nr	0.91	K-SADS-PL at Time 1	UCLA (8-item version)	17.62^b^ (5.28)	14.98^b^ (4.22)	Tests of difference and significance not reported.
Tracey and Gleeson (1998); Australia	ADHD-PI: 22 (18) ADHD-PHI: 19 (17) n-ADHD: 43 (21)	ADHDPI: 14.04 (2.14) ADHDPHI: 13.07 (2.66) n-ADHD: 14.29 (2.66)	nr	Score > 70 on PPVT-R	0.68	Previous diagnosis based on DSM-III	LACAYAS (study only used Peer-related loneliness subscale)	ADHD-PI: 33.9 (10.6) ADHD-PHI: 34.1 (9.2)	28.6 (7.5)	ADHD-PI and ADHD-PHI reported significantly more peer-related loneliness than non-ADHD, *p* < .05.

*Note*. Measures: ASRS = Adult ADHD Self-Report Scale (Kessler et al., 2005); CLSD = Children’s Loneliness and Dissatisfaction Scale (Asher & Wheeler, 1985); CLSD Hebrew adaptation (Margalit, 1991); CLSD Italian adaptation (Casiglia et al., 1998); COM = Comorbidity teacher’s report scale (Marzocchi et al., 2010); CRS = Conners Rating Scale (Conners, 1997); DBD = Disruptive Behavior Disorders Rating Scale (Pelham et al., 1992); Parent DBD structured interview (Pelham, 1994); DSM-5 = The Diagnostic and Statistical Manual of Mental Disorders - Fifth Edition (APA, 2013); DSM-IV = Diagnostic and Statistical Manual of Mental Disorders - Fourth Edition (APA, 1994); DSM-IV-TR = Diagnostic and Statistical Manual of Mental Disorders – Fourth Edition Text Revision (APA, 2000); ICD-10 = 10th revision of the International Statistical Classification of Diseases and Related Health Problems (World Health Organization, 2016); K-SADS = Schedule for Affective Disorders and Schizophrenia for School-Age Children (Kaufman et al., 1997); LACA = Loneliness and Aloneness Scale for Children and Adolescents (Marcoen et al., 1987); LACAYAS = The Loneliness Among Children and Young Adolescents Scale (Marcoen & Brumagne, 1985); PALs = The Perth A-loneness Scale (Houghton et al., 2014); PNDLS = Peer Network and Dyadic Loneliness Scale (Hoza et al., 2000); PPVT-R = Peabody Picture Vocabulary Test – Revised (Dunn & Dunn, 1981); SDAI = ADHD Rating Scale for Teachers (Marzocchi & Cornoldi, 2000); UCLA Loneliness Scale (Russell et al., 1978); UCLA Loneliness Scale Danish Version (Lasgaard, 2007); UCLA short version (D. Russell et al., 1980); UCLA Version 3 (D. W. Russell, 1996); UCLA Version 8-item version (Roberts et al., 1993); WISC-IV = Wechsler Intelligence Scale for Children – Fourth Edition (Wechsler, 2003); WPPSI-R = Wechsler Preschool and Primary Scale of Intelligence-Revised (Wechsler, 1990).

ADHD = attention-deficit/hyperactivity disorder; ADHD-PI = attention-deficit/hyperactivity disorder-predominantly inattentive; ADHD-PHI = attention-deficit/hyperactivity disorder-predominantly hyperactive; ASD = autism spectrum disorder; IQ = intelligence quotient; LD = learning disability; n-ADHD = non-attention-deficit/hyperactivity disorder; NDD = neurodevelopmental disorder; nr = not reported; ns = not significant; T1 = Time 1; TD = typical development.

a Studies shared overlapping sample.

b Relevant sub-sample obtained from author(s) as not reported in the original paper.

Overall, the sample size for participants with ADHD was 1,253 participants, and for the non-ADHD control group was 5,028 participants. Included in the count are two studies with overlapping samples (42 ADHD participants and 42 non-ADHD participants, Houghton et al., 2020; 76 ADHD participants 238 and non-ADHD participants, Houghton, Kyron, Lawrence et al., 2022). In terms of age, the range of the means in the overall samples of the studies (as some studies did not report age broken down by groups) was between 9.72 and 22.5 years old. There was a much higher representation of males in the ADHD sample in 10 studies, a higher representation of females in one study and the gender distribution for the ADHD sample was not reported in three studies. Few studies reported ethnicity (*n* = 4), with two reporting predominantly White/Caucasian samples, one reported all Greek, one predominantly Black and one with predominantly no ethnic affiliation. The ADHD participants in Al-Yagon’s (2009, 2016) studies were diagnosed with learning disabilities according to DSM-IV-TR and had average IQ and lower achievements on standardized testing than expected for age, schooling, and level of intelligence (APA, 2000).

#### Research question 1: Outcomes

Table 2 details the measures and outcomes for the studies comparing loneliness levels in ADHD and non-ADHD samples. Of the 17 studies, participants in the ADHD group either had previous formal diagnoses of ADHD or were diagnosed using clinician or self-/parent-reported DSM or ICD diagnostic codes or interviews in all but two studies (Capodieci et al., 2019; Koutra & Kokaliari, 2022). Capodieci et al. (2019) used informal interviews with teachers and parents, and a cut-off point on at least one of the two inattention and hyperactivity subscales of the teacher-report SDAI (Marzocchi & Cornoldi, 2000) to inform their ADHD grouping but the participants were not formally diagnosed with ADHD. Koutra and Kokaliari (2022) used a cut-off point of above 4 points on the ASRS, although some of their participants had a formal diagnosis of ADHD. For loneliness measures, the most commonly used measure were both the Children’s Loneliness and Social Dissatisfaction Scale (CLSD; Asher et al., 1984), including the different variations and adaptations, and the University of California, Los Angeles Loneliness Scale (UCLA; Russell et al. (1978)), including its different variations and adaptations, with five studies each. All studies used self-report questionnaires to measure loneliness in young people except for one which only measured parent-reported loneliness (Laslo-Roth et al., 2021). Heiman (2005) reported both self-reported loneliness in young people, and parent- and teacher-reported loneliness of the young people.

Of the 17 studies, nine studies reported significantly higher loneliness levels in the ADHD group compared to the non-ADHD group, four reported no significant difference between the groups, three studies did not test for significance, and one study (Deckers et al., 2017) reported significantly lower levels of loneliness in the ADHD group compared to the non-ADHD group in their adolescence group but no significant difference in their child group. Two of the studies that reported significantly higher loneliness levels in the ADHD group shared overlapping samples (Houghton et al., 2020; Houghton, Kyron, Lawrence et al., 2022). All studies that included parent-reported and teacher reported loneliness reported significantly higher loneliness scores compared to reports from parents and teachers of non-ADHD young people.

### Meta-Analysis for Research Question 1: Do Young People With ADHD Experience Greater Loneliness?

For the purposes of the meta-analysis, the two ages groups in Deckers et al. (2017) were combined. Two studies (Houghton et al., 2015, 2020) used the PALs, which included more than one subscale for loneliness, so the isolation loneliness subscale was chosen. Similarly, the peer-network loneliness subscale of the PNDLS in Al-Yagon (2016) was included in the meta-analysis. Additionally, Al-Yagon’s (2016) study compared the ADHD (with LD) group with a group without ADHD but with LD, and a TD group. The meta-analysis included the comparison between the ADHD-LD group and the TD group. The two ADHD subtype groups (ADHD-PI and ADHD-PHI) in Tracey and Gleeson (1998) were combined into an overall ADHD group for the meta-analysis.

**Figure 2.**
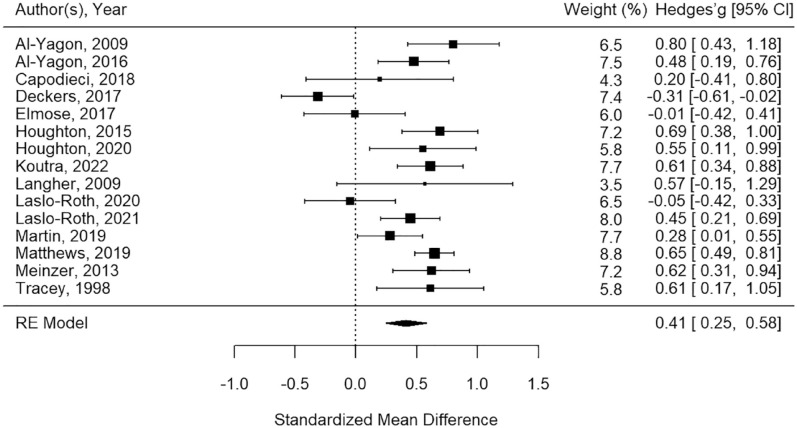
Forest plot for meta-analysis on comparison of loneliness levels between ADHD and non-ADHD group.

Figure 2 displays the standardized mean difference for the individual studies in the meta-analysis. Young people with ADHD reported significantly higher loneliness than young people without ADHD, with a small to medium weighted pool effect, Hedges’ *g* = 0.41, 95% CI [0.25, 0.58]; *z* = 4.83, *p* < .001.

In the meta-analysis, significant heterogeneity was found: *Q*(14) = 52.5, *p* < .001. The *I*^2^ suggested high heterogeneity, *I*^2^ = 75.1%, 95% CI [50.1%, 89.8%]. The influence of the studies included were assessed using “leave-one-out” analysis to demonstrate the heterogeneity contributed by the studies included. One study (Deckers et al., 2017) was identified as an outlier and contributed around 20% of the heterogeneity found between studies (see Table S2 in Supplementary Material for leave-one-out analysis). A sensitivity analysis was run, excluding the study, which produced a larger weighted pool effect (Hedges’ *g* = 0.48, 95% CI [0.35, 0.61]; *z* = 7.37, *p* < .001) and moderate heterogeneity, *I*^2^ = 52.64%, 95% CI [15.40%, 81.13%]; *Q*(13) = 26.43, *p* = .015.

Moderator analyses were also carried out to explore heterogeneity. Across the studies included in the meta-analysis, there were different loneliness measures used, none of which were used in enough studies to warrant a sub-group analysis; the most frequently used measure was the UCLA (*n* = 5) followed by the CLSD (*n* = 4). The following subgroup analyses were found to be non-significant: age group (child, adolescent), *Q*(1) = 0.00, *p* = 0.982; and, setting (community, clinical), *Q*(1) = 2.12, *p* = 0.146. For the age subgroup analysis, the category “both child and adolescent” was excluded as it had less than five studies. Additionally, meta-regression analyses indicated that the following were not significant moderators of effect-size: study quality, *Q*(1) = 2.12, *p* = 0.146; percentage of males in the ADHD group, *Q*(1) = 0.43, *p* = 0.511; and the mean age of the ADHD group, *Q*(1) = 0.002, *p* = 0.967. In total, 12 studies provided information on participant’s gender by group, and all but two studies provided the mean and standard deviations of the participants’ ages in the ADHD group.

**Figure 3.**
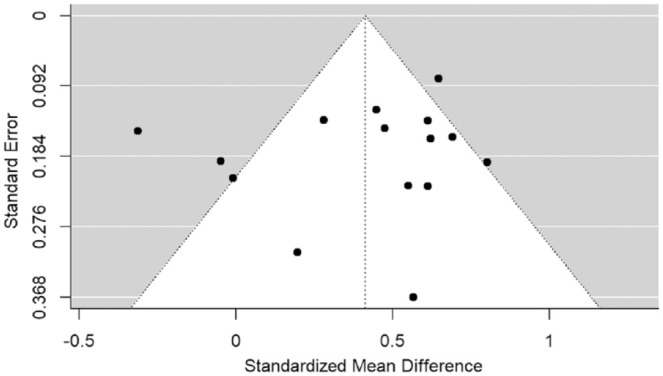
Funnel plot for meta-analysis on comparison of loneliness levels between ADHD and non-ADHD group.

The funnel plot (Figure 3) indicates potential asymmetry in the effect sizes of the studies, with four studies falling outside of the 95% confidence intervals, however, neither Egger’s regression test (*p* = 0.726) nor the Rank Correlation Test (*p* = 0.697) were significant, indicating that the likelihood of publication bias was low.

### Systematic Review for Research Question 2: What is the Association Between Loneliness and Mental Health Difficulties in Young People With ADHD?

#### Research question 2: Study characteristics

Table 3 summarizes the studies exploring the associations between loneliness and mental health difficulties in young people with ADHD. All studies were cross-sectional except for three which were longitudinal (Houghton, Kyron, Lawrence et al., 2022; Meinzer et al., 2013; Sciberras et al., 2022). There were 781 participants overall, including two overlapping studies (Houghton et al., 2020; Houghton, Kyron, Lawrence et al., 2022). The mean age of the young people ranged from 8.58 to 22.56 years old, although some studies did not specifically report the ages of the ADHD participants (Al-Yagon, 2016; Houghton, Kyron, Lawrence, et al., 2022; Meinzer et al., 2013). Two studies had an approximately equal distribution of genders (Al-Yagon, 2016; Li et al., 2016), four studies had a larger proportion of male participants (Deckers et al., 2017; Houghton et al., 2020; Sciberras et al., 2022; Smit et al., 2020), and two studies did not report gender specifically for the ADHD sample (Houghton, Kyron, Lawrence, et al., 2022; Meinzer et al., 2013). Three studies reported ethnicity for the overall sample, with two having predominantly White participants (Deckers et al., 2017; Smit et al., 2020) and one with predominantly Black participants (Meinzer et al., 2013). Of the eight studies, ADHD was previously formally diagnosed or was diagnosed using clinician or self-/parent-reported DSM or ICD diagnostic codes or interviews in all but one study which classified their ADHD participants using the Adult ADHD Self-Report Scale Chinese version (Gau et al., 1997; Kessler et al., 2005) if they scored higher than 17 on either of the subscales (Li et al., 2016). Six different loneliness measures were used across the eight studies included, with The Perth A-loneness Scale (PALS; (Houghton et al., 2014) and UCLA Loneliness Scale (UCLA; Russell et al., 1978) being the most frequently used loneliness measures with two studies each. As for measures of mental health, all the studies employed different measures except two studies who had overlapping samples (Houghton et al., 2020; Houghton, Kyron, Lawrence et al., 2022).

**Table 3.** Summary of Studies Examining the Association Between Loneliness and Mental Health Difficulties in ADHD (Research Question 2).

Authors (Year); Country	ADHD sample *n* (male)	ADHD age *M* (*SD*)	Study ethnicity	Study IQ	Study quality	ADHD measure	Loneliness measure	Mental health measure	Key findings
Al-Yagon (2016); Israel	91 (41)	Nr Overall sample: 15.94 (0.70) No sig. diff between ages of groups	nr	Normal level: WISC-IV	0.82	Previous ADHD diagnosis DSM-IV-TR using clinical interview, computerized tests. Diagnosis validated by document check.	PNDLS	YSR (Hebrew adaptation; Externalizing/Internalizing Syndrome scales)	Peer-network loneliness in the LD-ADHD group is significantly correlated with internalizing behaviors (*r* = .57, *p* < .001) but not externalizing behaviors (*r* = .15, *p* = nr). Peer-dyadic loneliness in the LD-ADHD group is not significantly correlated with internalizing behaviors nor externalizing behaviors (*p* = nr).
Deckers et al. (2017); Netherlands	76 (54)	11.79 (2.48)	Predominant Caucasian	Severe cognitive (IQ < 70) or language impairments excluded.	0.82	Previous diagnosis by a multidisciplinary team using multiple sources (i.e., interviews, observations, assessments)	LACA (only relationships with peer subscale was used in the study)	SCARED-71 (parent-reported)	In ADHD, children-reported loneliness is partially correlated (corrected for gender) with parent-reported social anxiety (*r* = .35, *p* < .001). In ASD and control group, loneliness not correlated with social anxiety.
Houghton et al. (2020)^a^; Australia	42 (31)	13.01 (2.0)	nr	nr	0.73	Previously diagnosed by a pediatrician or child psychiatrist as meeting DSM-IV-TR or DSM-5 criteria.	PALs	CDI:SR	Isolation loneliness is correlated with depression in ADHD (*r* = .67, *p* < .001). Friendship loneliness is correlated with depression in ADHD (*r* = −.68, *p* < .001). Together, friendship and isolation loneliness fully mediated the relationship between ADHD and depressive symptoms. The total indirect effect of an ADHD diagnosis on depressive symptoms, through friendship related loneliness and isolation, was statistically significant (*B* = 2.29, β = .24, *p* < .001).
Houghton, Kyron, Lawrence et al. (2022)^a^; Australia	76 (nr)	nr Total NDD: 13.52 (1.44)	nr	nr	0.77	Previous diagnosis as meeting DSM-IV-TR/DSM-5 criteria for ADHD.	PALs	CDI:SR; SDQ	At baseline, correlations between isolation loneliness and: CDI:SR Depression (*r =* .53, *p* < .001) SDQ Internalizing (*r* = .67, *p* < .001) SDQ Externalizing (*r* = .38, *p* < .001)^b^
Li et al. (2016); China	73 (39)	22.56 (3.19)	nr	nr	0.77	ASRS (Chinese version), > 17 on either subscale classified as ADHD group	UCLA (version 3)	CIAS-R: ≥ 64 was classified as the Internet addiction group	Both loneliness in ADHD scores (*r* = .54, *p* < .01) and non-ADHD (*r* = .52, *p* < .01) is correlated with higher internet addiction. In a hierarchical linear regression, loneliness, impulsiveness and behavioral inhibition were significant predictors of internet addiction in ADHD.
Meinzer et al. (2013); USA	T1: *40 (nr) Overall: 1,709 (803)	T1: nr Overall: 16.6 (1.2)	T1: nr Overall: 62% Black, 36% white, 1% Hispanic, 1% ‘other’	nr	0.91	K-SADS-PL; DSM-III-R	UCLA (8-item version)	SCID-NP; LIFE	*In a univariate regression, T1 loneliness significantly predicted MDD onset in ADHD (*r* = .49, *p* < .01). In the multivariate regression, ADHD remained a significant predictor of MDD onset even after loneliness (*p* = ns), coping skills, new onset psychiatric disorders, psychiatric disorders, gender, life stress, academic impairment were added as predictors.
Sciberras et al. (2022); Australia	212 (162)	10.59 (3.1)	nr	nr	0.90	Prior diagnosis of ADHD	CRISIS (Parent-reported loneliness subscale)	CRISIS (Sad/depressed/unhappy and anxious/nervous subscales)	*2 months before COVID: loneliness and sad/depressed (*r* = .39) loneliness and worried/anxious (*r* = .35). May 2020/COVID: loneliness and sad/depressed (*r* = .56) loneliness and worried/anxious (*r* = .47).
Smit et al. (2020); Canada	213 (147)	8.58 (1.55)	70% White, 5% Pacific Islander/Asian, 1% Latino/Hispanic, 1% Afro-Canadian/Black, 16% multi-racial, 6% not reported	IQ below 75 on WASI/short form WISC-IV excluded	0.90	Prior diagnosis of ADHD; parent-rated Child Symptom Inventory (at least 4 hyperactivity and inattention), parent K-SADS-PL Exclusion: ID, ASD, severe MH e.g., suicidality	CLSD	Parent endorsement of child meeting DSM-IV-TR criteria for a relevant disorder (internalizing or externalizing) on the K-SADS, in addition to second informant (child or teacher).	*Loneliness is associated with child internalizing comorbidities (*r* = .32, *p* < .001) but not child externalizing comorbidities (*r* = .05, *p* = .468). Bivariate correlations: Loneliness was associated with more internalizing comorbidities for boys (*r* = .28, *p* < .01) and girls (*r* = .40, *p* < .01). However, externalizing comorbidities was associated with more loneliness in boys (*r* = .17, *p* < .05), but not girls. After controlling for gender and age, loneliness was associated with more internalizing disorders (β = .31, *p* < .01) but not externalizing disorders (β = .03, *p* = ns).

*Note.* Measures: ASRS = Adult ADHD Self-Report Scale (Kessler et al., 2005); ASRS Chinese version (Gau et al., 1997); CBCL = Child Behavior Checklist (Achenbach, 1999); CDI:SR = The Children’s Depression Inventory Short Version (Kovacs, 2004); CIAS-R = The Revised Chen Internet Addiction Scale (Chen et al., 2016); CLSD = Children’s Loneliness Scale (Asher et al., 1984); CRISIS = CoRonavIruS Health Impact Survey (Nikolaidis et al., 2021); DSM-III-R = Diagnostic and Statistical Manual of Mental Disorders – Third Edition Revised (APA, 1987); DSM-IV-TR = Diagnostic and Statistical Manual of Mental Disorders – Fourth Edition Text Revision (APA, 2000); DSM-5 = Diagnostic and Statistical Manual of Mental Disorders – Fifth Edition (APA, 2013); K-SADS-PL = Kiddie-Schedule for Affective Disorders and Schizophrenia-Present and Lifetime Version (Kaufman et al., 1997); LACA = Loneliness and Aloneness Scale for Children and Adolescents (Marcoen et al., 1987); LIFE = Longitudinal Interval Follow-Up Evaluation (Keller, 1987); PALs = The Perth A-loneness Scale (Houghton et al., 2014); PNDLS = Peer Network and Dyadic Loneliness Scale (Hoza et al., 2000); SCID-NP = Structured Clinical Interview for Axis I DSM-IV Disorders (Gorgens, 2011); SDQ = The Strengths and Difficulties Questionnaire (Goodman, 1997); TRF = Teacher’s Report Form (Achenbach & Rescorla, 2001); UCLA Loneliness Scale-Version (D. W. Russell, 1996); UCLA Version 8-item version (Roberts et al., 1993); WASI = Wechsler Abbreviated Scale of Intelligence (Wechsler, 2011); WISC-IV = Wechsler Intelligence Scale for Children – Fourth Edition (Wechsler, 2003); YSR = Youth Self-Report 11-18 (Achenbach, 1991); YSR Hebrew adaptation (Zilber et al., 1994). ADHD = Attention Deficit/Hyperactivity Disorder; ASD = Autism Spectrum Disorder; LD = Learning Disability; MDD = Major Depressive Disorder; NDD = Neurodevelopmental Disability; nr = not reported; ns = not significant; T1 = Time 1; TD = Typical Development.

a Studies shared overlapping sample.

b Relevant sub-sample obtained from author(s) as not reported in the original paper.

#### Research question 2: Outcomes

##### Externalizing

Houghton, Kyron, Lawrence et al. (2022) found significant correlations between loneliness and externalizing symptoms (e.g., conduct difficulties) on the SDQ. In a sample of clinically diagnosed children with ADHD aged 6 to 11 years old, Smit et al. (2020) found significant correlations between loneliness and externalizing disorder (parent-endorsed ODD or CD on the K-SADS) in males but not in females. After controlling for gender, age, and internalizing disorders, externalizing disorders were not associated with loneliness. In Al-Yagon’s (2016) study, neither peer-network loneliness nor peer-dyadic loneliness were significantly associated with externalizing behavior (e.g., delinquency and aggressiveness) on the YSR.

##### General Internalizing

Two studies found significant correlations between loneliness and internalizing behaviors or symptoms (Houghton, Kyron, Lawrence et al., 2022; Smit et al., 2020). Smit et al.’s (2020) study found that internalizing disorders on the K-SADS, which covered endorsement of any anxiety or depressive disorder, had significant positive correlations with loneliness after controlling for gender, age and externalizing disorders. Houghton, Kyron, Lawrence et al. (2022) used the internalizing scale on the SDQ, which covered emotional and peer problems, and found that internalizing behaviors had significant positive correlations with isolation loneliness and friendship loneliness. One study found that internalizing symptoms (e.g., withdrawal and anxiety/depression) on the YSR was only significantly associated with peer-network loneliness but not peer-dyadic loneliness (Al-Yagon, 2016).

##### Depression

Three studies explored the association between loneliness and depression (Houghton et al., 2020; Houghton, Kyron, Lawrence et al., 2022; Meinzer et al., 2013). Houghton et al. (2020)’s study explored to what extent loneliness explained the relationship between ADHD diagnosis and depressive symptoms. They found, in adolescents with ADHD, significant positive correlations between isolation loneliness and depression, and negative correlations between friendship loneliness (having reliable, supportive friends) and depression. Furthermore, after controlling for age and gender, friendship and isolation related loneliness fully mediated the association between depression and ADHD symptoms. Houghton, Kyron, Lawrence et al. (2022), which shared an overlapping sample with Houghton et al. (2020), also found significant positive correlations between isolation loneliness and depression. One study reported small-to-medium correlations (significance unreported) between loneliness and sad/depressed symptoms before COVID and an increased effect size during COVID (May 2020) in children and adolescents (Sciberras et al., 2022). Meinzer et al. (2013) found that loneliness levels in mid-adolescence predicted the onset of Major Depressive Disorder in early adulthood in ADHD. This study also looked at whether ADHD status, loneliness, and other predictors were significantly associated with MDD onset in adolescents and found that ADHD diagnosis remained as a significant predictor for MDD onset after controlling for loneliness, gender, other psychiatric disorders, life stress, coping skills, and academic impairment.

##### Anxiety

Deckers et al. (2017) found significant partial correlations (corrected for gender) between loneliness and social anxiety in children and adolescents with ADHD. They did not find similar associations between loneliness and social anxiety in children and adolescents with ASD, nor in the typical development control group. Another study examined the impact of COVID-19 social restrictions on young people aged 5 to 17 years old and measured symptoms before and during COVID (Sciberras et al., 2022). They reported small-to-medium correlations between loneliness and worried/anxious symptoms in young people before COVID and an increased effect size of the correlation between loneliness and worried/anxious symptoms during COVID (May 2020), but the significance of the correlations was unreported.

##### Addiction

Higher levels of loneliness in adolescents and young adults have been found to be correlated with higher internet addiction scores, but this pattern was also seen in the non-ADHD group (Li et al., 2016). Loneliness, impulsivity, and behavioral inhibition were all significant predictors of internet addiction in ADHD in a hierarchical linear regression (Li et al., 2016).

## Discussion

This review carried out the first meta-analysis and systematic review to explore whether young people with ADHD experienced greater loneliness compared to young people without ADHD. The combined effect size for the significant increased loneliness in the ADHD group was small-to-medium (Hedges’ *g* = 0.41). There was a high level of heterogeneity between the studies and non-significant effects of age and gender of the ADHD group, study quality, and recruitment setting. For the second research question, which reviewed the association between loneliness and mental health difficulties in young people with ADHD, most studies reported an association between loneliness and mental health difficulties including general internalizing behaviors and symptoms, anxiety, depression, externalizing behaviors and symptoms, and addiction.

### Loneliness Levels in Young People With ADHD

The meta-analysis for the first question provides an important contribution to our understanding of loneliness in young people with ADHD. That is, current state of the literature included in this study suggests that young people with ADHD report significantly higher levels of loneliness compared to those without ADHD. This is consistent with findings of individuals with ADHD perceiving their friendships as having fewer positive and more negative features (Normand et al., 2011) and being less satisfied with their social networks (Grygiel et al., 2018), suggesting that there is an awareness of a gap in their social relationships. One possible factor that might explain the increased loneliness in young people with ADHD compared to their peers could be the greater peer rejection and social difficulties experienced (Hoza et al., 2005; McQuade & Hoza, 2008). Indeed, other research shows that loneliness follows peer rejection and peer difficulties (Ladd & Troop-Gordon, 2003; Pedersen et al., 2007). It is also possible that the loneliness experience in ADHD encompasses more than social and peer difficulties and may also relate to feeling different. Adults with ADHD expressed feeling loneliness in suffering in relation to their disability, illness and care, and feeling different, which persisted from childhood (Björk et al., 2017).

While the meta-analysis found overall increased loneliness levels in young people with ADHD compared to their peers without ADHD, there were some studies included that found no difference or decreased levels of loneliness in the ADHD group. This is interesting in light of some theories positing that people with ADHD may have lower loneliness levels due to a self-perceptual bias which protects them from feeling lonely, and thus masking associations between ADHD and loneliness (Hoza et al., 2005; Martin et al., 2019). For example, when children with ADHD were compared with those without ADHD, but had similar social skills difficulties, children with ADHD viewed themselves as more popular and interpersonally competent compared to teacher reports while children without ADHD had similar ratings to the teacher reports (Capodieci et al., 2019). Martin et al. (2019) reported that ADHD diagnosis was associated more strongly with loneliness when social self-perceptual bias was controlled for. It is possible that a self-perceptual bias may affect loneliness reports and that this review’s findings are an underestimation. More research is required to examine to loneliness in young people with ADHD as there were some limitations (expanded below) of the studies included that could have impacted the results.

The meta-analysis found a high level of heterogeneity, which warranted further moderator and meta-regression analyses. However, there was a lack of significant findings in the analyses. Some factors that may have contributed to this could be, for example, overlaps between ages in the child and adolescent groups. Similarly, the limited variability in age of the sample, rather than an even distribution throughout the age range, may have impacted the meta-regression. The lack of significance when comparing the research setting could perhaps partly be due to some of the community settings being special educational or inclusive schools which may have obscured some of the difference in severity of ADHD symptoms. The proportion of males in the ADHD sample in the studies were much larger compared to females, potentially impacting the meta-regression. It is also possible that there may not be a gender difference in loneliness in young people with ADHD, similar to the general population (Maes et al., 2019). The quality of the studies did not significantly explain the between-study heterogeneity in the meta-analysis, though many of the studies had similar quality scores of around 0.73 to 0.82. Other factors that were not analyzed as a moderator may have contributed to the heterogeneity, such as, the type of loneliness measure or the ADHD subtype.

### Loneliness and Mental Health Difficulties in Young People With ADHD

Most studies reviewed found a significant positive association between loneliness with different mental health difficulties in young people with ADHD, including general internalizing behaviors and symptoms, anxiety, depression, and internet addiction. It should be noted that there were few studies included in this systematic review and most of the studies did not look at the same mental health difficulties so caution should be used when interpreting the results. The general finding that loneliness is associated with mental health problems is in line with the wealth of studies in the general population, including in adults and young people (Heinrich & Gullone, 2006; Loades et al., 2020), in young people with pre-existing mental health problems (Hards et al., 2022), and also in other neurodevelopmental populations (Hymas et al., 2022; Kwan et al., 2020). Additionally, Al-Yagon (2016) reported that different forms of loneliness were differentially associated with internalizing and externalizing symptoms, which is consistent with conceptualizations of loneliness as being multi-dimensional (Hoza et al., 2000). In the general population, studies have shown that loneliness predicts depressive and anxious symptoms (S. L. Lee et al., 2021; Wang et al., 2018), with some showing bidirectional associations (Cacioppo et al., 2006). There small number of studies included in this review were mostly cross-sectional, so our understanding of the directionality of the association between loneliness and mental health problems in young people with ADHD is still limited. More research, including longitudinal studies, is required to replicate the few studies available and to better understand how loneliness may vary with different mental health difficulties and the causality between loneliness and mental health difficulties.

### Strengths and Limitations of the Included Studies

There were multiple different aspects of the studies included in this paper that should be taken into consideration when interpreting the findings. The measurement and reporting of the demographics in the studies were sometimes inconsistent and only reported information for the overall sample, rather than in the specific subgroups. There was a much larger proportion of males than females in the studies included, and most studies were from Western countries, limiting the generalizability of this review. The methodological quality of the studies included in this review was generally good (see Tables 2 and 3). However, there were aspects that could have introduced bias such as ethnicity and socio-economic status that were not controlled for (Qualter et al., 2021; Visser & El Fakiri, 2016).

Some methodological concerns relate to the assessments and measures used across studies. In terms of ADHD categorization, most of the studies included previous diagnosis of ADHD and were scarce on details of how the diagnosis was made. A recommended “gold standard” for ADHD diagnosis includes many aspects including history taking and interviews, rating scales, and behavioral observations which can be resource intensive (Gualtieri & Johnson, 2005). The ADHD diagnoses in the studies included in this review may reflect how ADHD diagnoses are carried out in the real world and provide important information. Future studies would benefit from administering both interviews and rating scales to diagnose or validate prior ADHD diagnoses. There were also inconsistencies regarding ADHD subtype, medication status, and comorbidities reporting.

Due to the many different types of loneliness measures used across different studies, it is difficult to compare loneliness levels and prevalence between studies, in addition to interpreting whether the elevated loneliness levels experienced by young people with ADHD in this review are clinically relevant or not (Nicolaisen & Thorsen, 2014; Surkalim et al., 2022). Future studies could benefit from using multiple loneliness measures, including direct and indirect measures, to aid with comparability (Office for National Statistics, 2018).

### Strengths and Limitations of this Review

This systematic review’s search strategy was based on PRISMA guidelines, was pre-registered on PROSPERO for transparency, excluded papers that were not peer-reviewed, and focused on loneliness/perceived social isolation in young people who were categorized as having ADHD in the studies included. A quarter of the screening was replicated by an independent rater, and the quality assessment for all papers included was carried out by two independent raters, bolstering the reliability of the screening and quality appraisal. The search strategy used for this review was also broad and the search was carried out in multiple databases (including reviewing references of relevant studies), so the likelihood of missing relevant studies was low. The differences between the studies were also analyzed in the moderator analysis and meta-regression as opposed to having restrictive inclusion criteria. The small number of studies included in the moderator and meta-regression analyses decreases the confidence in the interpretation of the results.

This review only investigated loneliness in ADHD in studies that categorized their participants into ADHD groups and excluded studies that examined how loneliness is associated with ADHD symptoms. Dichotomous categorization of ADHD can sometimes miss out less severe presentations of ADHD which may have affected this review (Ramtekkar et al., 2010). To reduce potential bias and increase the representativeness, it is recommended that future reviews investigate the association between loneliness and ADHD symptom severity.

### Clinical Implications

This review highlights that loneliness may be an important problem in ADHD and clinicians should be aware of and assess the potential for elevated loneliness in this population. Given the possible mental health problems associated with loneliness in ADHD, reducing loneliness may not only decrease the distress but may also positively impact other aspects of mental health. Currently, individuals with ADHD who experience relationship difficulties are often offered social skills training (Mikami et al., 2014), which have limited effectiveness on social skills, emotional competencies, and general behavior (Storebø et al., 2019). These interventions also fail to consider the wider context which maintains the peer difficulties and the distressing experience of loneliness in the young person (Mikami et al., 2014). There exists interventions that lower loneliness in young people generally, such as those focusing on picking up a new hobby, and learning social and emotional skills (Eccles & Qualter, 2021) but they do not distinguish between transient and prolonged loneliness, and were not ADHD specific. The loneliness experienced in ADHD may be more prolonged perhaps due to both their social functioning difficulties which often persists into adolescence and adulthood (S. S. Lee et al., 2011; Wehmeier et al., 2010) and feeling different due to the hardships faced due to their ADHD (Björk et al., 2017), so social skills and emotion management training which is helpful for transient loneliness may be insufficient (Qualter et al., 2015). A better understanding of the experience of loneliness in young people with ADHD, including what contributes to their loneliness, may aid in developing loneliness interventions targeted for this population. Additionally, it could be beneficial to focus on improving other aspects linked to loneliness such as increasing close friendships (Al-Yagon, 2016; Kingery et al., 2011; Smit et al., 2020).

### Conclusions

The findings in this study highlight the importance of understanding loneliness in this population as young people with ADHD report significantly higher levels of loneliness compared to their non-ADHD peers, and loneliness in young people with ADHD is associated with a range of mental health difficulties. Despite some of the limitations, this review provides support for considering loneliness as part of the wide-ranging social-emotional difficulties that young people with ADHD are at higher risk of experiencing. As it is recommended that interventions aimed at individuals with ADHD should target the different aspects of their difficulties (National Institute for Health and Clinical Excellence, 2018), more resources should be focused on loneliness as a separate construct from social isolation and peer difficulties. ADHD is a complex neurodevelopmental condition, which is associated with higher risks of comorbidities and adverse outcomes so early identification and treatment of loneliness may be especially important in this population.

## Supplemental Material

sj-docx-1-jad-10.1177_10870547241229096 – Supplemental material for Loneliness in Young People with ADHD: A Systematic Review and Meta-Analysis Supplemental material, sj-docx-1-jad-10.1177_10870547241229096 for Loneliness in Young People with ADHD: A Systematic Review and Meta-Analysis by Angelina Jong, Clarissa Mary Odoi, Jennifer Lau and Matthew J.Hollocks in Journal of Attention Disorders
