# Supplementary material for: Loneliness in Young People with ADHD: A Systematic Review and Meta-Analysis
Source: J Atten Disord. 2024 Feb 23;28(7):1063–81. doi: 10.1177/10870547241229096 (PMC11016212; doi:10.1177/10870547241229096)
Supplement: sj-docx-1-jad-10.1177_10870547241229096 – Supplemental material for Loneliness in Young People with ADHD: A Systematic Review and Meta-Analysis [file sj-docx-1-jad-10.1177_10870547241229096.docx]

**Supplementary Material**

**Table S1**

**Quality Appraisal Ratings according to QualSyst (Kmet et al., 2004)**

| Study | Q1 | Q2 | Q3 | Q4 | Q5 | Q6 | Q7 | Q8 | Q9 | Q10 | Q11 | Q12 | Q13 | Q14 | Score | |
| --- | --- | --- | --- | --- | --- | --- | --- | --- | --- | --- | --- | --- | --- | --- | --- | --- |
| Al‐Yagon (2009) | 1 | 2 | 1 | 2 | N/A | N/A | N/A | 1 | 1 | 2 | 2 | 1 | 2 | 2 | 0.77 |  |
| Al-Yagon (2016) | 2 | 2 | 1 | 1 | N/A | N/A | N/A | 1 | 2 | 2 | 2 | 1 | 2 | 2 | 0.82 |  |
| Capodieci et al. (2018) | 2 | 2 | 1 | 1 | N/A | N/A | N/A | 2 | 1 | 1 | 1 | 1 | 1 | 2 | 0.68 |  |
| Deckers et al. (2017) | 2 | 2 | 1 | 2 | N/A | N/A | N/A | 1 | 1 | 2 | 2 | 1 | 2 | 2 | 0.82 |  |
| Elmose & Lasgaard (2017) | 2 | 1 | 1 | 2 | N/A | N/A | N/A | 1 | 1 | 2 | 2 | 1 | 1 | 2 | 0.73 |  |
| Heiman (2005) | 1 | 2 | 1 | 2 | N/A | N/A | N/A | 1 | 1 | 1 | 1 | 1 | 1 | 2 | 0.64 |  |
| Houghton et al. (2015) | 2 | 2 | 1 | 2 | N/A | N/A | N/A | 1 | 1 | 2 | 2 | 2 | 1 | 2 | 0.82 |  |
| Houghton et al. (2020) | 2 | 2 | 1 | 1 | N/A | N/A | N/A | 1 | 1 | 2 | 1 | 2 | 2 | 1 | 0.73 |  |
| Houghton et al. (2022) | 2 | 2 | 1 | 1 | N/A | N/A | N/A | 1 | 2 | 2 | 1 | 2 | 1 | 2 | 0.77 |  |
| Koutra & Kokaliari (2022) | 2 | 2 | 1 | 1 | N/A | N/A | N/A | 1 | 2 | 2 | 2 | 1 | 1 | 2 | 0.77 |  |
| Langher et al. (2009) | 2 | 2 | 1 | 1 | N/A | N/A | N/A | 1 | 1 | 1 | 1 | 1 | 2 | 2 | 0.68 |  |
| Laslo-Roth et al. (2020) | 2 | 2 | 1 | 2 | N/A | N/A | N/A | 1 | 2 | 2 | 2 | 1 | 1 | 2 | 0.82 |  |
| Laslo-Roth et al. (2021) | 2 | 2 | 1 | 1 | N/A | N/A | N/A | 1 | 2 | 2 | 2 | 1 | 2 | 2 | 0.82 |  |
| Li et al. (2016) | 2 | 1 | 1 | 1 | N/A | N/A | N/A | 2 | 1 | 2 | 2 | 1 | 2 | 2 | 0.77 |  |
| Martin et al. (2019) | 2 | 2 | 1 | 1 | N/A | N/A | N/A | 2 | 2 | 2 | 2 | 1 | 1 | 1 | 0.77 |  |
| Matthews et al. (2019) | 1 | 2 | 2 | 1 | N/A | N/A | N/A | 2 | 2 | 2 | 2 | 1 | 2 | 2 | 0.86 |  |
| Meinzer et al. (2013) | 2 | 2 | 1 | 1 | N/A | N/A | N/A | 2 | 2 | 2 | 2 | 2 | 2 | 2 | 0.91 |  |
| Sciberras et al. (2020) | 2 | 2 | 1 | 1 | N/A | N/A | N/A | 2 | 2 | 2 | 2 | N/A | 2 | 2 | 0.90 |  |
| Smit et al. (2020) | 2 | 2 | 2 | 2 | N/A | N/A | N/A | 1 | 2 | 2 | 1 | N/A | 2 | 2 | 0.90 |  |
| Tracey & Gleeson (1998) | 2 | 1 | 1 | 2 | N/A | N/A | N/A | 1 | 1 | 2 | 1 | 1 | 1 | 2 | 0.68 |  |

Q1. Question / objective sufficiently described? Q2. Study design evident and appropriate? Q3. Method of subject/comparison group selection or source of information/input variables described and appropriate? Q4. Subject (and comparison group, if applicable) characteristics sufficiently described? Q5. If interventional and random allocation was possible, was it described? Q6. If interventional and blinding of investigators was possible, was it reported? Q7. If interventional and blinding of subjects was possible, was it reported? Q8. Outcome and (if applicable) exposure measure(s) well defined and robust to measurement / misclassification bias? Means of assessment reported? Q9. Sample size appropriate? Q10. Analytic methods described/justified and appropriate? Q11. Some estimate of variance is reported for the main results? Q12. Controlled for confounding? Q13. Results reported in sufficient detail? Q14. Conclusions supported by the results?

**Table S2**

**Leave-one-out analysis for meta-analysis (Research Question 1)**

| Study | *g* [95% CI] | | *SE* | *z*-value | *p-*value | *Cochrane Q* | *Cochrane Q p*-value | tau^2^ | *I^2^* (%) |
| --- | --- | --- | --- | --- | --- | --- | --- | --- | --- |
| Al‐Yagon (2009) | 0.39 [0.22, 0.56] | | 0.09 | 4.42 | < .001 | 50.5 | < .001 | 0.07 | 74.8 |
| Al-Yagon (2016) | | 0.41 [0.23, 0.59] | 0.09 | 4.41 | < .001 | 53.95 | < .001 | 0.08 | 76.77 |
| Capodieci et al. (2018) | | 0.42 [0.25, 0.60] | 0.09 | 4.75 | < .001 | 53.28 | < .001 | 0.08 | 76.93 |
| Deckers et al. (2017) | | 0.48 [0.35, 0.61] | 0.07 | 7.37 | < .001 | 26.43 | 0.01 | 0.03 | 52.64 |
| Elmose & Lasgaard (2017) | | 0.44 [0.27, 0.61] | 0.09 | 5.09 | < .001 | 49.07 | < .001 | 0.07 | 74.65 |
| Houghton et al. (2015) | | 0.39 [0.22, 0.57] | 0.09 | 4.37 | < .001 | 51.55 | < .001 | 0.08 | 75.48 |
| Houghton et al. (2020) | | 0.40 [0.23, 0.58] | 0.09 | 4.46 | < .001 | 53.78 | < .001 | 0.08 | 77.12 |
| Koutra & Kokaliari (2022) | | 0.40 [0.22, 0.57] | 0.09 | 4.35 | < .001 | 52.49 | < .001 | 0.08 | 75.79 |
| Langher et al. (2009) | | 0.40 [0.23, 0.58] | 0.09 | 4.59 | < .001 | 53.88 | < .001 | 0.08 | 77.08 |
| Laslo-Roth et al. (2020) | | 0.45 [0.28, 0.61] | 0.09 | 5.24 | < .001 | 46.76 | < .001 | 0.07 | 73.37 |
| Laslo-Roth et al. (2021) | | 0.41 [0.23, 0.59] | 0.09 | 4.4 | < .001 | 53.97 | < .001 | 0.09 | 76.27 |
| Martin et al. (2019) | | 0.42 [0.24, 0.60] | 0.09 | 4.6 | < .001 | 52.23 | < .001 | 0.08 | 76.22 |
| Matthews et al. (2019) | | 0.39 [0.21, 0.57] | 0.09 | 4.3 | < .001 | 46.66 | < .001 | 0.08 | 72.47 |
| Meinzer et al. (2013) | | 0.40 [0.22, 0.57] | 0.09 | 4.37 | < .001 | 52.8 | < .001 | 0.08 | 76.21 |
| Tracey & Gleeson (1998) | | 0.40 [0.22, 0.58] | 0.09 | 4.44 | < .001 | 53.45 | < .001 | 0.08 | 76.87 |
